# Role of gut microbiota‐derived metabolites on vascular calcification in CKD

**DOI:** 10.1111/jcmm.16230

**Published:** 2020-12-27

**Authors:** Li Yin, XiaoXue Li, Sounak Ghosh, Changming Xie, Jie Chen, Hui Huang

**Affiliations:** ^1^ Department of Cardiology The Eighth Affiliated Hospital Sun Yat‐sen University Shenzhen China; ^2^ Department of Radiation Oncology Sun Yat‐sen Memorial Hospital Sun Yat‐sen University Guangzhou China

**Keywords:** chronic kidney disease, gut microbiota‐derived metabolites, vascular calcification

## Abstract

The interaction between gut microbiota and the host has gained widespread concern. Gut microbiota not only provides nutrients from the ingested food but also generates bioactive metabolites and signalling molecules to impact host physiology, especially in chronic kidney disease (CKD). The development of CKD, accompanied by changed diet and medication, alters the gut flora and causes the effect in distant organs, leading to clinical complications. Vascular calcification (VC) is an actively regulated process and a high prevalence of VC in CKD has also been linked to an imbalance in gut microbiota and altered metabolites. In this review, we focused on gut microbiota‐derived metabolites involved in VC in CKD and explained how these metabolites influence the calcification process. Correcting the imbalance of gut microbiota and regulating microbiota‐derived metabolites by dietary modification and probiotics are new targets for the improvement of the gut‐kidney axis, which indicate innovative treatment options of VC in CKD.

## 1 INTRODUCTION

Chronic kidney disease (CKD) is a global public health problem. The Global Burden of Disease study estimated that nearly 697 million persons worldwide had reduced the estimated glomerular filtration rate (eGFR) in 2016. It had increased by 70% since 1990.^1^ Cardiovascular disease (CVD) is the leading cause of early death in the setting of CKD.^2^ In addition, vascular calcification (VC) is a risk factor for major adverse cardiovascular events (MACEs), especially in patients with CKD.^3^, ^4^

Gut microbiota is a broader ecological community that influences the body's normal physiological function and disease susceptibility through the interaction between metabolism and host.^5^ Simenhoff et al, through endoscopy, first demonstrated in the 1970s that gut microbiota was significantly altered in both CKD and non‐CKD patients. In CKD patients, aerobic and anaerobic bacteria were intensely colonized in the duodenum and jejunum.^6^ In addition, decreased Lactobacillus and increased Enterobacteriaceae had been observed.^7^ Moreover, faecal analysis of dialysis patients revealed a decreased level of short‐chain fatty acid butyrate.^8^ Once gut flora destroys the intestinal epithelial barrier and releases such detrimental metabolites into circulation, inflammation, oxidative stress and direct active pathways induced by the metabolites eventually lead to the development and progression of VC.^9^ Accumulating evidence demonstrated that gut microbiota might play an essential role in VC in CKD patients.^10^, ^11^ Therefore, this review intended to provide an overview of gut microbiota‐derived metabolites on the facilitation of VC and to propose new thoughts based on the interference of gut microbiota‐derived metabolites to retard VC in CKD patients.

## 2 VC IN CKD

The vascular wall is composed of three differently structured layers from the periphery to the lumen of the vessel. Blood vessels contain two primary cell types, endothelial cells (ECs) and vascular smooth muscle cells (VSMCs), which exert essential functions to maintain vascular homeostasis. Being different from ECs, VSMCs are not terminally differentiated and preserve their plasticity. VC can develop in the intimal and medial layers of arteries. Alternatively, medial calcification is characterized by VSMCs' transformation into osteoblast‐like cells and is more common in CKD patients.^12^ Overproduction of reactive oxygen species (ROS) in VSMCs is related to vascular dysfunction, which is a risk factor for CKD patients. VC is an active biological process associated with hydroxyapatite crystallisation in the vascular wall. The decline of inhibitors such as Matrix Gla Protein (MGP), Gla‐rich protein (GRP), osteoprotegerin (OPG), bone morphogenetic protein 7 (BMP‐7) and the increase of calcification inducers lead to more extensive VC in the CKD population.^13^, ^14^ Furthermore, uremic toxins, calcium and phosphate metabolism dysfunction may directly influence VSMCs' physiological function, leading to irregular senescence, proliferation and migration of VSMC, ultimately leading to VC.

## 3 THE GUT MICROBIOTA IN CKD

The human gastrointestinal tract is inhabited with 100 trillion different microbes, including bacteria (Lactobacillus), viruses (primarily phage), fungi, archaea and so on.^15^ There are two characteristics of gut microbiota in healthy adults; one is taxonomical diversity, such as Bacteroidetes, Firmicutes, Actinobacteria, Proteobacteria and Verrucomicrobia all be seen in the healthy gut.^16^ The other is functional diversity, such as enhancing the host against enteral pathogens, modulating systemic immunity. In the body, approximately 70% of the immune cells reside in the gut, cutting down bacterial dissemination and producing vitamins and essential metabolites that are not synthesized by the host.^17^ A study amongst end‐stage renal disease (ESRD) patients found high phyla Firmicutes, Proteobacteria and Actinobacteria, and a decrease in Lactobacilli, Roseburia and Phytoalexin.^18^

**FIGURE 1:**
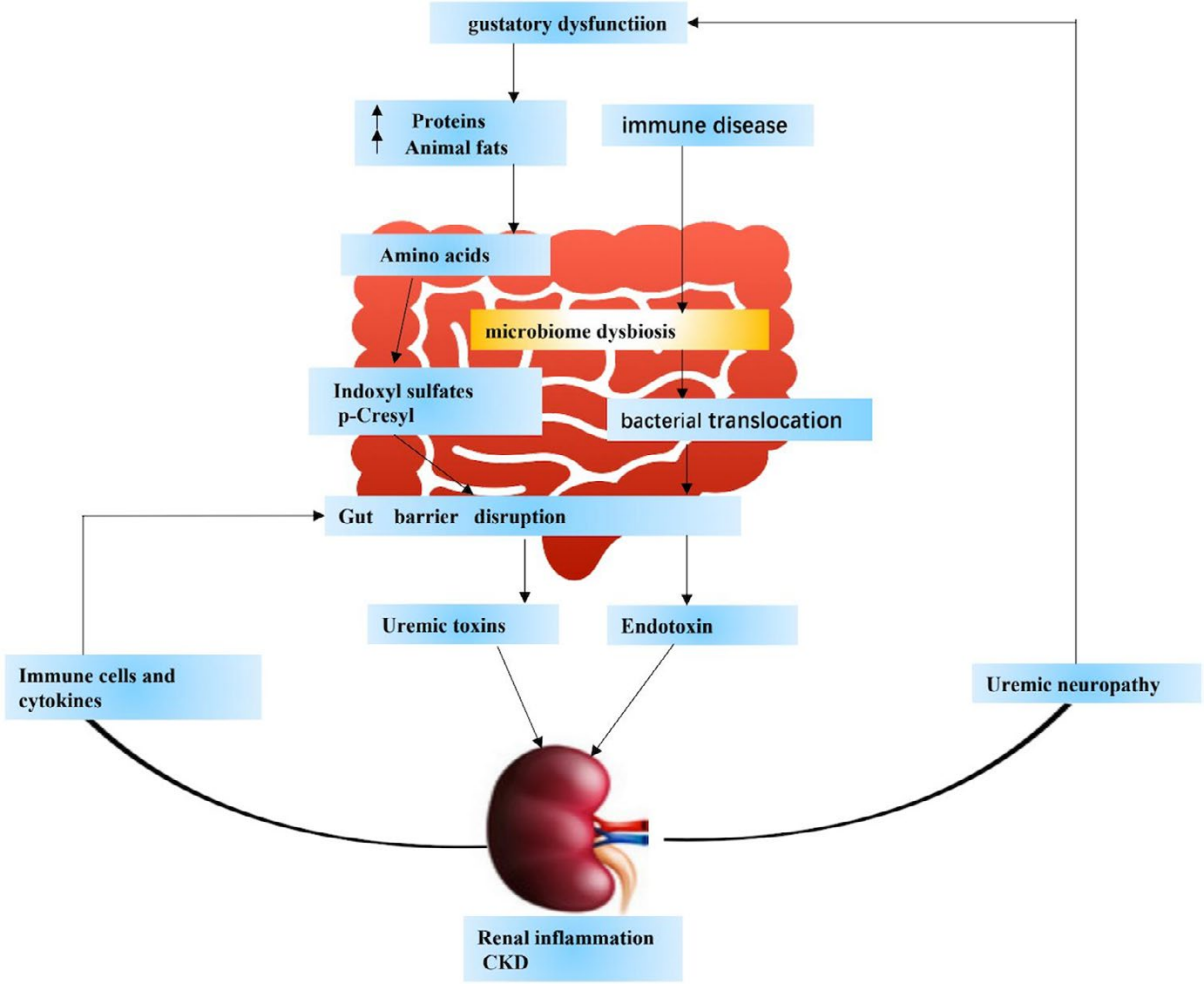
The kidney plays an important role in nutritional homeostasis. Base on Chronic kidney disease causes kidney damage, Increased susceptibility to malnutrition injury. In CKD, a reduction in renal filtering capacity results in the deposition and accumulation of waste products in the blood, which eventually develops into uremia. In addition, complications of uremia include uremic neuropathy, which can contribute to gustatory dysfunction leads to an imbalanced diet. Dysbiosis caused by an imbalanced diet (for example, a diet high in protein and animal fat) leads to excessive production and accumulation of p‐cresol and indoxyl sulfate in the gut. This accumulation destroys the intestinal barrier, thereby increasing the permeability of the intestine. Therefore, it can cause kidney damage (like inflammation of the kidneys). Metabolite causes activation of immune cells and factors, and continuous destruction of the intestinal barrier. This process into a vicious circle

Currently, several studies proposed the role of the gut‐kidney axis (Figure 1). For example, excessive protein intake in the diet can cause intra‐glomerular hypertension, which leads to kidney ultrafiltration and glomerular damage. Long‐term high protein intake may lead to CKD. In CKD patients, a reduction in renal filtering capacity results in the deposition and accumulation of waste products in the blood, which eventually develops into uremia. Uremia can lead to malnutrition, which leads to an imbalance of intestinal flora and abnormal metabolites, increased intestinal permeability. Finally, kidney damage is aggravated again.^19^ In another study with CKD patients, it was demonstrated that the reduction infiltration capacity of the kidneys led to the deposition and accumulation of toxic waste products in the blood.^7^ The genome of the gut microbiome contains 3.3 million genes. It is 150 times the human genome. Recently, a study has shown that dual‐omics (metagenomic and metabolomics) data can reveal the connections between gut microbes and circulating metabolites perturbed in CKD. In the early stage of CKD, microbial genes to secondary bile acid biosynthesis were differentially abundant. In the advanced stage that lipid metabolism and lipopolysaccharide biosynthesis were enriched. However, the research lacks a replication cohort.^20^

## 4 INFLUENCE OF DIET ON GUT MICROBIOTA

Diet strongly affects human health. Most of the beneficial effects are obtained by modulating gut microbiome composition. According to the dominant bacterial system type, the gut microbiota of adults can be divided into two main types. Both intestinal types are closely related to long‐term diet. The main bacterial population of intestinal type 1 is Bacteroides, which mainly metabolizes proteins, while gut type 2 is mainly glycolytic chlorella.^21^ Previously, animal studies reported that intake of a high‐unsaturated fat‐rich diet would increase Actinobacteria (Lactobacillus and Streptococcus) and Verrucomicrobia. Probiotics intake, such as cultured milk products and yogurt, is a source of ingestible microorganisms.^22^ Diet containing high fat can increase Lipopolysaccharides (LPS) translocation.^23^ Common food like fruits, vegetables and tea are all rich in polyphenols.^24^ Probiotics and Polyphenols both enhance Bifidobacterium and Lactic acid‐producing bacteria and reduce enteropathogenic bacteria.^25^ The renal diet (low potassium, low phosphorus), which is lacking in plant fibre, can lead to the overgrowth of bacteria with harmful metabolites like uremic toxins.^26^ A study reported that older adults with CKD had a higher taste sensation for phosphate‐containing salts. Hyperphosphatemia can accelerate VSMCs transdifferentiating and directly participate in the deposition of the calcium‐containing osteoid matrix in vascular media. Therefore, CKD with gustatory dysfunction can develop metabolism perturbation and contribute to uremic neuropathy. If the gustatory function in CKD patients can be preserved, food intake can be improved and reduce the production of uremic solutes, leading to a lower risk of VC.^27^ Therefore, diet can adjust the type of gut microbiota and metabolites. In CKD patients, more attention should be paid to the impact of diet in VC.

## 5 THE RELATIONSHIP BETWEEN GUT MICROBIOTA‐DERIVED METABOLITES AND VC IN CKD

**FIGURE 2:**
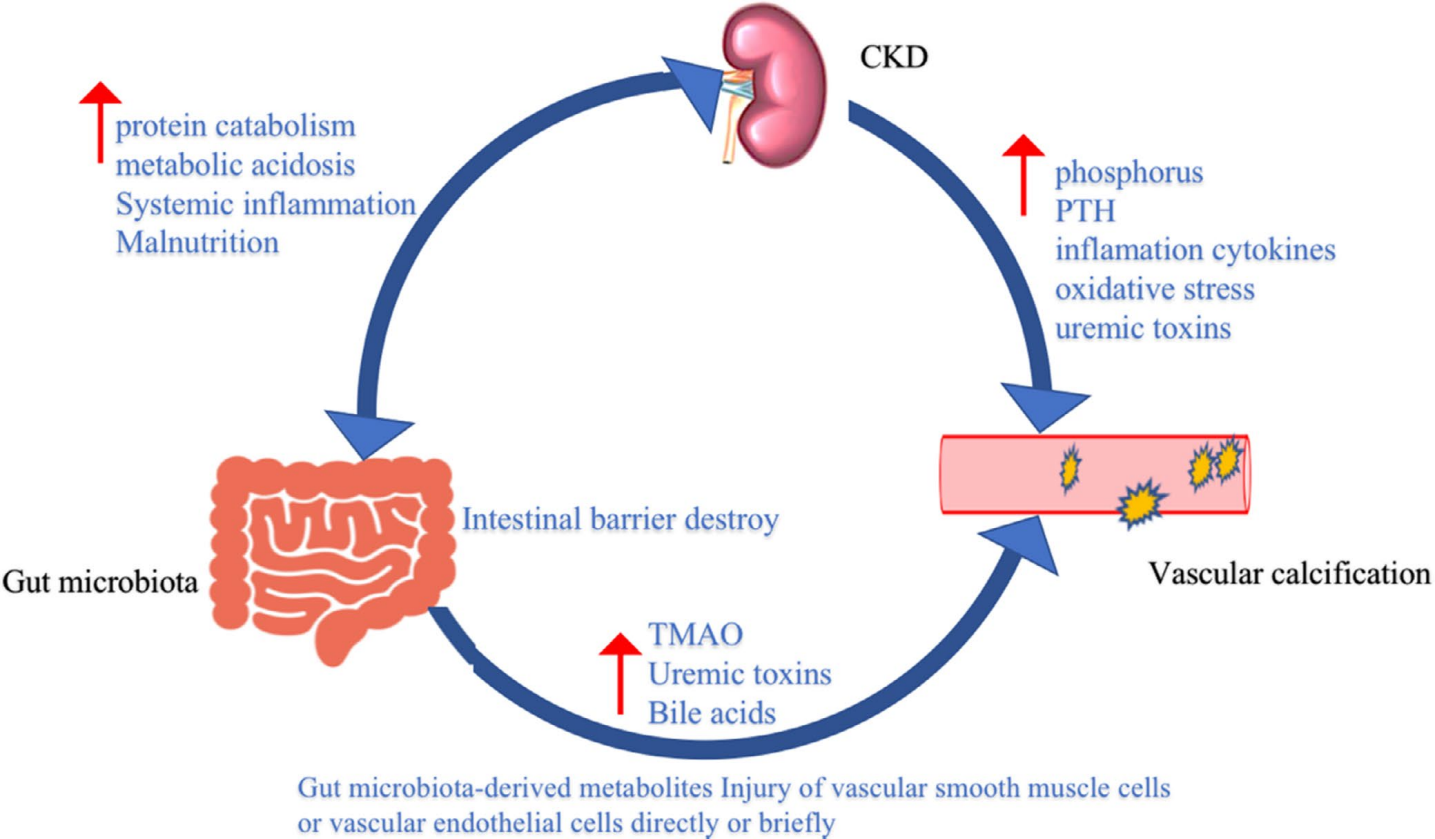
In chronic kidney disease (CKD) patients, decreased renal function leads to decreased glomerular filtration rate (GFR), increased proteinuria and uremic toxins, and damage to glomeruli and tubulointerstitial. Also cause metabolic acidosis, accelerated protein catabolism, resulting in malnourished patients. Malnutrition also leads to an imbalance of intestinal homeostasis, which is characterized by increased mucosal inflammation, increased intestinal permeability and abnormally increased gut microbiota‐derived metabolites like (p‐cresol, indoxyl sulfate and Trimethylamine N‐oxide (TMAO)). It can directly or indirectly affect vascular smooth muscle cells or vascular endothelial cells and induce vascular calcification. And high phosphorus caused by chronic kidney disease, parathyroid hormone (PTH), inflammatory cytokines, oxidative stress and uremic toxins can induce vascular calcification

Altered gut microbiota and metabolites in CKD are believed to be involved in the VC process.^28^ LPS and bacterial DNA could directly induce inflammation and immune response, leading to end‐organ damage.^29^ Gut microbiota‐derived metabolites such as uremic toxins, trimethylamine N‐oxide (TMAO), bile acid are all associated with VC in CKD by regulating vascular phenotype, oxidative stress and epigenetics.^30^, ^31^ The following is a detailed introduction of gut microbiota‐derived metabolites on vascular calcification (Figure 2).

### 5.1 Uremic toxins

Gut microbiota‐derived metabolites of amino acids are uremic toxins, including indole‐3 acetic acid, indoxyl sulfate (IS) and p‐cresyl sulfate (PCS), which translocate into the bloodstream and cause extensive oxidative stress‐induced damage to the kidneys. Uremic toxins can lead to endothelial dysfunction, vascular senescence, vascular inflammation in CKD patients. It is supporting the link between uremic toxins and vascular dysfunction.^32^ Silvia D et al demonstrated that uremic toxins impaired the autophagic flux leading to endothelial dysfunction.^33^ Generally, gut bacteria metabolize tryptophan into indole, further exchanged into indoxyl sulfate in the liver after intestinal absorption.^34^ Gut bacteria also metabolize aromatic amino acids into tyrosine phenylalanine and p‐cresol, which are bio‐transformed by sulfotransferase into PCS in the liver.^35^, ^36^ In CKD patients, the production of uremic toxins (such as IS, PCS) by bacteria increases.

IS, a critical protein‐bound uremic toxin, can be described as a significant risk factor of VC in CKD patients.^37^ Research has shown that uremic toxins (mainly Pi) are responsible for the high prevalence of VC in the CKD population.^38^ In a healthy human beings, IS concentration ranges from 10 to 130 mg/day. But excessive IS induces the production of free radicals in both renal cells and VSMC through oxidative stress and inflammation to cause tissue injury.^39^ In CKD patients, clinical evidence has shown that IS plasma levels are associated with pulse wave velocity, ankle‐brachial index, which are markers of arteriosclerosis and aortic calcification. IS can induce vascular inflammation through the delta‐like (DII) 4‐Notch signalling pathway.^40^ Moreover, IS can induce the proliferation, osteogenic differentiation and senescence of VSMC by regulating the Mitogen‐activated protein kinase (MAPK) pathway, p21‐p27‐p53 pathway and PI3K/Akt/NF‐κB pathway.^41^ IS promotes VSMC calcification through the secretion of IL‐8 by endothelial cells in the presence of inorganic phosphate. In addition, it enhances the cytosine‐guanine CpG hypermethylation of klotho and epigenetic modification of klotho to promote the process of VC in CKD. It was shown to up‐regulate the expression of intercellular cell adhesion molecule‐1 (ICAM‐1) and monocyte chemotactic protein (MCP‐1) in a vascular endothelial cell through ROS‐induced activation of nuclear factor‐κβ (NF‐κβ).^42^ Also, IS induced methyltransferase‐like (METTL14)‐dependent N6‐methyladenosine (m6A) to regulate VC.^43^

PCS is a prototype protein‐bound molecule.^44^ PCS's concentration ranges between 2.8 ± 1.7 mg/L and 6.6 ± 3.7 mg/L in healthy human plasma. However, PCS is significantly increased in end‐stage renal disease (ESRD) patients (21.8 ± 12.4 mg/L and 106.9 ± 44.6 mg/L).^45^ PCS induced inflammatory factors that triggered monocyte‐endothelial cell interaction and incriminated oxidative stress in human VSMCs.^46^ In addition, the effect of PCS on renal injury has been reported. Sun et al demonstrated that PCS was activated by the renal renin‐angiotensin‐aldosterone system and then induced epithelial‐mesenchymal transition, contributing to kidney injury.^47^

Moreover, a study has demonstrated that IS and PCS both could potentially induce endothelial dysfunction and distinct calcification in the arteries of CKD rats.^31^ On the one hand, IS generates stress in ECs and induces premature senescence via increasing macro‐vesicles (MVs) release. On the other hand, PCS also promotes EMV release, which causes the dysregulation of vascular homeostasis. At the beginning of the development of VC, MVs are involved in the inflammatory response of VSMCs. The MVs might be useful to develop as biomarkers and therapeutic tools for preventing CKD at risk of developing VC.^48^, ^49^ Further in vivo experiment is still needed to illustrate its role. AST‐120 is an oral carbon adsorbent. It can absorb IS and PCS precursors, reducing serum IS and PCS levels in CKD patients, which may be beneficial to reduce VC.^50^ Clinical trials demonstrated that symbiotic (pre‐and probiotic) therapy decreased serum PCS and modified stool microbiome.^51^ Thus, it could be a novel therapy for the management of VC in CKD patients.

**FIGURE 3:**
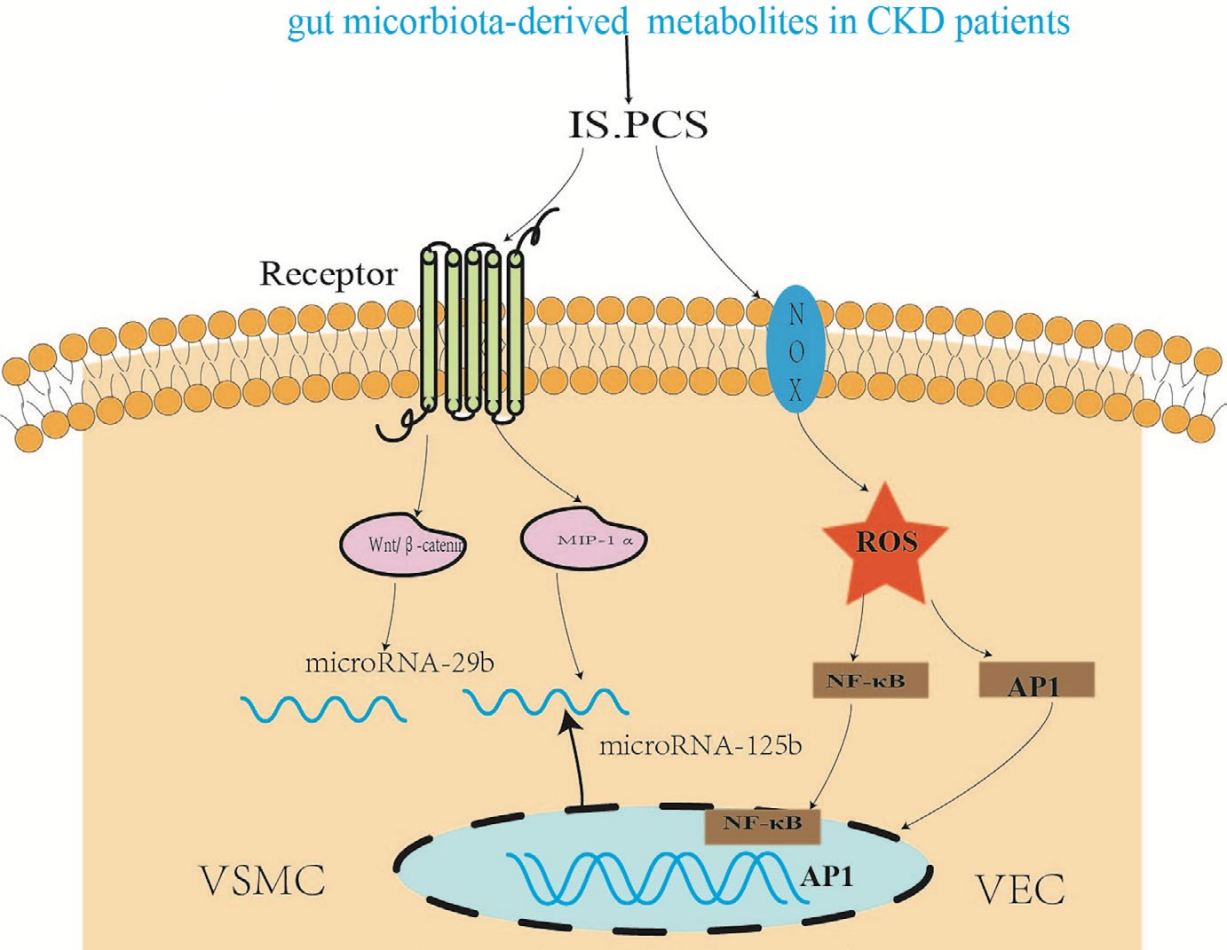
In patients with chronic kidney disease (CKD), the effect of gut microbiota‐derived metabolites indoxyl sulfate (IS) and p‐cresyl sulfate (PCS) on vascular smooth muscle cell (VSMC). IS down‐regulated miR‐29b and activated Wnt/β‐catenin signalling to induce vascular activation. IS induced reactive oxygen species (ROS) also promote the activation of Nuclear factor‐κB (NF‐κB) and activating protein 1 (AP‐1) pathways, increase inflammation and damage the endothelial cell

Recent studies have shown that microRNAs (miRs) as epigenetic regulators are involved in uremic toxins‐induced vascular calcification. MicroRNAs are small non‐coding RNAs and regulate the protein expression without affecting the gene sequence. Besides, microRNAs are potential biomarkers of VC induced by uremic toxins, such as miR‐223, miR‐125 and miR‐143.^52^, ^53^ In cultured endothelial cells (ECs), IS up‐regulated miR‐92a to activate the inflammasome in ECs and enhanced vascular inflammation.^54^ IS also down‐regulated miR‐29b and activated Wnt/β‐catenin signalling to induce vascular activation.^55^ In conclusion, IS and PCS are considered harmful vascular toxins and promote VC in CKD patients. MVs and miRNAs might be therapeutic targets to prevent vascular disease in CKD (Figure 3).

### 5.2 Trimethylamine N‐oxide (TMAO)

Trimethylamine N‐oxide is a product of the gut microbiome. Choline and phosphatidylcholine are catalysed into trimethylamine (TMA) in intestinal microbiota (such as phosphatidylcholine, betaine and I‐carnitine), which is further oxidized as TMAO in the human liver.^56^

**FIGURE 4:**
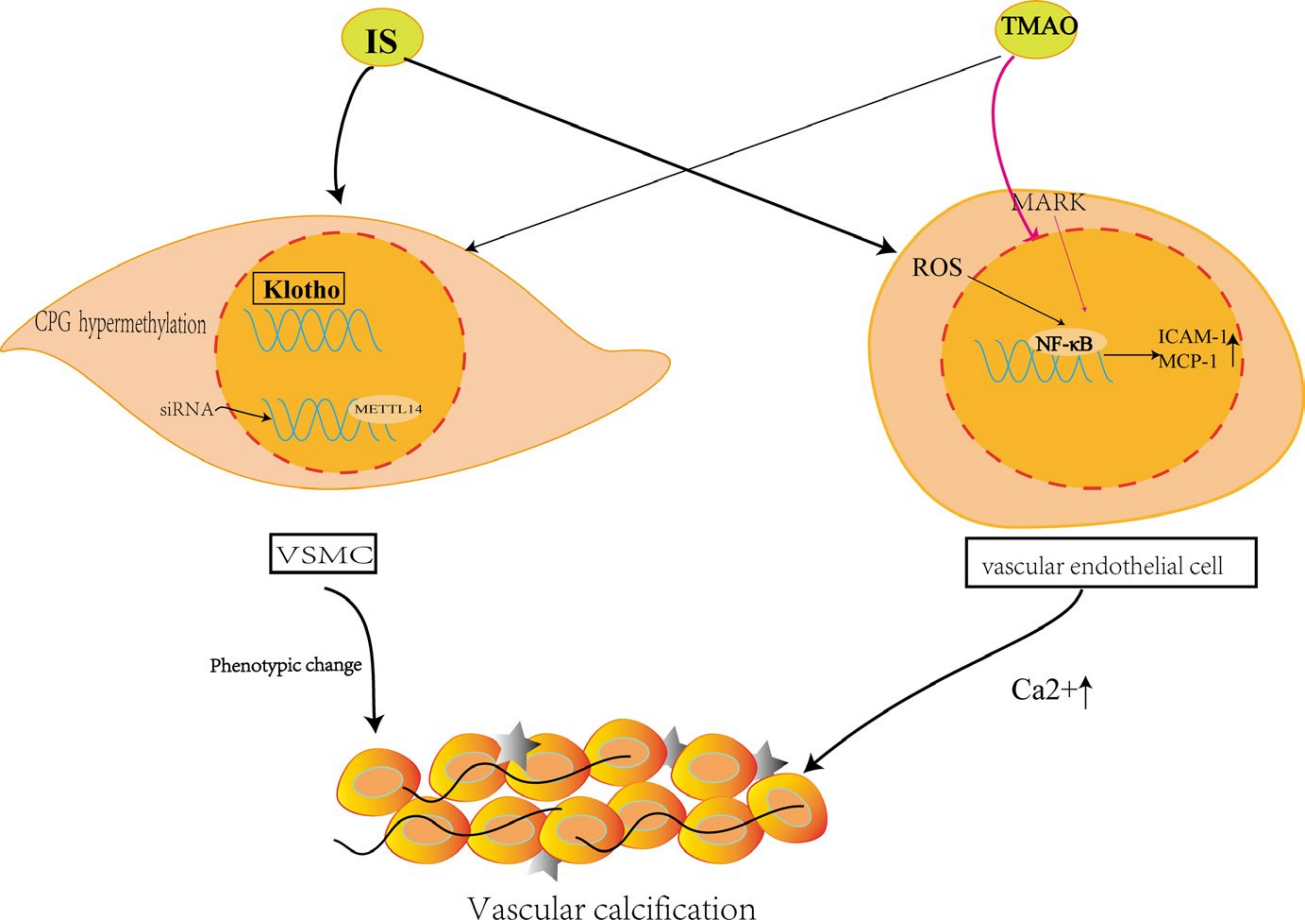
IS enhances the CpG hypermethylation of Klotho and epigenetic modification of klotho to promote the process of VC in CKD and induced methyltransferase‐like (METTL14) ‐dependent N6‐methyladenosine (m6A) regulated vascular calcification in VSMC.TMAO activates endothelial cell mitogen‐activated protein kinase (MAPK) and vascular smooth muscle cell (VSMC) through nuclear factor‐κB (NF‐κB) pathway, leading to inflammatory gene expression and augmenting Ca2+ release from intracellular stores

In vitro experiments showed that TMAO promoted VC only in a calcifying medium, explaining that high calcium and phosphate were critical for TMAO‐induced VC. In vivo study demonstrated that TMAO promoted VC in CKD rats with high calcium/ phosphorus (Ca/P) diet.^57^ Besides, TMAO was shown to increase the expression of pro‐inflammatory genes such as interleukin‐18 (IL‐18), interleukin‐6 (IL‐6) and interleukin‐1β (IL‐1β) adhesion molecules and chemokines.^58^ Besides, TMAO increased the expression of pro‐inflammatory genes such as IL‐18, IL‐6 and IL‐1β adhesion molecules and chemokines.^59^ In human umbilical vein endothelial cells (HUVEC) and aortas from ApoE knockout mouse, TMAO also induced oxidative stress and nucleotide‐blinding domain, leucine‐rich‐containing family, pyrin domain‐containing‐3 (NLRP3) activation signals, releasing of inflammatory cytokines.^59^, ^60^, ^61^ TMAO activated endothelial cell mitogen‐activated protein kinase (MAPK) and VSMC through NF‐κβ pathway, leading to inflammatory gene expression and augmenting Ca^2+^ release from intracellular stores (Figure 4).

In summary, the elevated TMAO level in CKD is a considerable promoter of VC. Several approaches are being explored to reduce TMAO levels, like oral broad‐spectrum antibiotics or promoting the growth of bacteria that use TMAO as matrix and baicalin.^61^, ^62^, ^64^

Recently, the Pretest‐posttest study showed that a diet supplemented with β‐glucan was potentially efficient in lowering serum concentrations of TMAO in patients with CKD.^30^ But whether these methods are able to prevent VC still needs further research.

### 5.3 Bile acids

Bile acids are amphipathic molecules that have two types. One is primary bile acids and another is secondary bile acids. Primary bile acids produced by cholesterol in the liver are processed into secondary bile acids by gut microbiota. Bile acid metabolism affects host metabolism through the regulation of the cholesterol cholestero7‐α hydroxylase (CYP7A1) and G protein‐coupled receptor (TGR5, GPBAR1).^65^, ^66^ In CKD patients, circulate bile acids present a high level. When bile acids are perturbed, primary bile acid is decreased, and secondary bile acid, deoxycholic acid (DCA) are increased.

DCA is directly toxic to VSMCs. A clinical trial proved that high DCA is an independent risk factor for VC in CKD patients.^67^ DCA can induce mineralisation and osteogenic differentiation of VSMCs by regulating endoplasmic reticulum (ER) stress. Emerging data implicate the role of ER stress as a new mechanism for VC.^68^ Primary bile acids (BAs) increase the colon Retinoid‐related orphan receptor) ROR acids + Regulatory T (Treg) cells improve host susceptibility. Tregs can modulate both innate and adaptive immune responses to suppress VC in CKD.^69^ Researchers have discovered that the genome‐wide biliary network interaction between intestinal bacteria and the host can control the host's immune homeostasis.^70^ Besides, Bas and their nuclear receptor, such as farnesoid X receptor (FXR), are found in macrophages and vasculature. Importantly, activation of FXR was shown to reduce VC in the CKD model.^71^

### 5.4 Lipopolysaccharide (LPS)

LPS, also called endotoxin, is a specific type of gut microbiota‐derived metabolite. It is a component of the outer membrane of gram‐negative bacteria. If the intestinal barrier is impaired by bacterial translocation and gut microbiota disorder, LPS can enter the circulation through the gut wall and induce a systemic inflammatory response.^72^, ^73^

Evidence suggested that pro‐inflammatory cytokines such as IL‐6 induced VSMC mineralisation and osteogenic transition, playing a pivotal role in the progression of VC.^74^ The research demonstrated that IL‐18 also contributed to VC in pro‐inflammatory conditions.^75^ Inflammatory cytokines increased the expression of bone morphogenetic protein 2 (BMP2) and reduced MGP expression, further promoting VC formation in VSMCs.^76^ Inflammatory responses are essential regulators in the development of VC in CKD patients. A study found in ESRD patients that great phyla Firmicutes, Proteobacteria and Actinobacteria have decreased in Lactobacilli, Roseburia and Phytoalexin. Furthermore, intestinal microbiome dysbiosis also led to bacterial translocation that colon wall inflammation follows with the destruction of the enteric epithelial barrier, which leads to LPS and translocation of bacterial DNA into the bloodstream, triggering a state of persistent systemic inflammation in CKD patients.^18^, ^77^ Recently, having evidence that LPS and translocation of bacterial DNA can promote Pi‐induced calcification and osteoblastic differentiation in human aortic smooth muscle cells (HASMCs) through TLR9/NF‐κβ/BMP‐2signalling.Furthermore, Toll‐like receptor 4 (TLR4) and Toll‐like receptor 9 is un‐regulated in VSMCs aggravated inorganic Pi induced VC in CKD patients. Sanchis P et al demonstrated that level of inflammatory markers correlated with VC, while blocking ataxia‐telangiectasia mutated (ATM)‐mediated DNA damage signalling reduced inflammation and calcification in CKD patients.^78^, ^79^, ^80^

### 5.5 Short‐chain fatty acids (SCFAs)

Short‐chain fatty acids (SCFAs) are another significant gut microbiota‐derived metabolite. SCFAs are produced through the fermentation of dietary fibres by anaerobic gut bacteria in the caecum and proximal colon.^81^ SCFAs are mainly composed of acetate, propionate and butyrate. Butyrate is primarily metabolized by the colonic commensal bacteria, which regulates cell growth and differentiation.^82^ SCFAs can be used to maintain the gut barrier and inhibit pathogenic microbe proliferation in acidic PH conditions.^83^ It is also mediated by G protein‐coupled receptors GRP419 (FFAR3) and GRP43 (FFAR2), predominantly expressed in the immune cell. SCFAs also can contribute to improving vascular phenotypes.^84^, ^85^

## 6 POTENTIAL THERAPEUTIC STRATEGIES AGAINST VC

The intervention of gut microbiota‐derived metabolites is a potential strategy to reduce calcification in CKD patients. Resveratrol is a dietary polyphenol compound. It has anti‐inflammatory, antioxidative properties. Recently, studies also showed that resveratrol is a phytoalexin and scavenger for many free radicals. Resveratrol can decreased plasma TMAO and regulated sirtuin‐1 (Sirt‐1) and nuclear factor‐E2‐associated factor 2 (Nrf2) signalling pathway to ameliorate VC vascular calcification. ^86^ Besides, Resveratrol can reverse the effect of IS. Also, resveratrol was shown to inhibit IS‐activated aryl hydrocarbon receptor (AHR) and regulate VE‐cadherin and permeability‐induced increase in Src activation.^87^ Resveratrol could protect rat VMSCs against oxidative injury in VC. A range of studies has highlighted that interfering with some intestinal flora metabolites is beneficial for vascular suppression (Table 1).^88^, ^89^, ^90^, ^91^, ^92^, ^93^, ^94^, ^95^ S‐equol is produced naturally in the gut by the bacterial biotransformation of daidzein, a soy isoflavone. A number of studies suggested that S‐equol can inhibit vascular remodelling in the protective vascular system. S‐equol can protect vasculature against cardiovascular diseases. Thus, S‐equol should have an important role in the field of vascular research.^96^ It is thus proposed that the use of oral iron supplements might improve the gut microbiome. However, iron supplements can lead to a decreased abundance of Lactobacillus and Bifidobacterium species.^97^ Recently, many ongoing experiments have been focusing on the diet being the most significant modifiable factor capable of changing gut microbiota.^98^ The development of diet (such as probiotics) as a primary management option to regulate the intestinal flora is pivotal to retard the development and progression of VC.

**TABLE 1:** Change in gut microbiota‐derived metabolites to treat VC

	Gut microbiota	Function	Reference number
Anthocyanins(blackberries)	LPS	Inhibitory gram‐negative bacteria	90.91
PCA	Produced by intestinal explanation	Reduce miRNA‐10b expression (miRNA‐10b directly harmful regulates human)	92
Probiotics	SCFAs Bifidobacterium	Increase SCFAs, Bifidobacterium, lactic acid bacteria	93.94
Probiotics	Roseburia intestinalis	Anti‐inflammatory response by regulating Treg cells.	95
Antibiotics	TMAO	Reduce TMAO	62
Buckwheat honey	*S. aureus* and *E. coli*	Inhibitory activity	96
Gallic acid	Harmful bacteria	Clostridium histolyticum	97

Abbreviations: *E. coli*, *Escherichia coli*; LPS, Lipopolysaccharide; PCA, Protocatechuic acid; SCFAs, Short‐chain fatty acids; TMAO, Trimethylamine N‐oxide; VC, vascular calcification.

## 7 CONCLUSION

Gut microbiota is closely related to human health. The gut microbiota and metabolites have significant effects on CKD patients especially with the complication of VC. In this review, we focused on intestinal microbiota‐derived metabolites such as uremic toxins, TMAO and SCFAs in VC. The multi‐factorial mechanism of VC suggests that the intervention of gut microbiota‐derived metabolites could be one of the important strategies to inhibit VC in CKD. There are existing methods like dietary or pharmacological intervention affecting genomics, intestinal absorption, gut‐kidney axis to reduce the production of harmful substances in the intestine and might further to improve VC. Thus, considering the significance of gut microbiota, future research should further explore the direct relationship between gut microbiota‐derived metabolites and VC.

## CONFLICT OF INTEREST

The authors declare that they have no conflict of interest.

## AUTHOR CONTRIBUTIONS

**Li Yin:** Conceptualization (equal); Data curation (equal); Formal analysis (equal); Methodology (equal); Writing‐original draft (lead); Writing‐review & editing (equal). **xiao xue Li:** Formal analysis (equal); Resources (equal); Writing‐original draft (equal); Writing‐review & editing (equal). **Sounak Ghosh:** Writing‐review & editing (lead). **Changming Xie:** Resources (equal). **Jie Chen:** Supervision (equal); Visualization (equal). **Hui Huang:** Conceptualization (lead); Formal analysis (lead); Methodology (lead); Resources (lead); Supervision (lead); Writing‐original draft (equal); Writing‐review & editing (lead).
